# Prevalence of human T-cell lymphotropic virus in blood donor screening at the Blood Center: a descriptive study, Mato Grosso, 2018-2021

**DOI:** 10.1590/S2237-96222026v35e20240799.en

**Published:** 2025-02-16

**Authors:** Pennsylvania Marinho Borralho, Eduarda Pavan, Francisco Kennedy Scofoni Faleiros de Azevedo, Cor Jesus Fernandes Fontes, Anmony Borralho de Figueiredo, Wagner Izidoro de Brito, Fernanda Rizzieri Yamanaka, Ruberlei Godinho de Oliveira

**Affiliations:** 1 Hemocentro de Mato Grosso, Secretaria de Estado de Saúde de Mato Grosso, Cuiabá, MT, Brazil; 2 Universidade Federal de Mato Grosso, Laboratório de Virologia, Cuiabá, MT, Brazil; 3 Universidade Federal de Mato Grosso, Cuiabá, MT, Brazil; 4 Secretaria de Estado de Saúde de Mato Grosso, Cuiabá, MT, Brazil; 5 Hospital Universitário Júlio Müller, Cuiabá, MT, Brazil

**Keywords:** Deltaretrovirus, Prevalence, Demography, Blood Banks, Seroepidemiologic Studies., Deltaretrovirus, Prevalencia, Demografía, Bancos de Sangre, Estudios Seroepidemiológicos.

## Abstract

**Objectives:**

To describe the prevalence of human T-lymphotropic virus types 1 and 2 (HTLV-1/2) infection among blood donors at the Mato Grosso Blood Center between January 2018 and August 2021, as well as to report the demographic characteristics and coinfections identified in infected donors.

**Methods:**

: This was a descriptive, retrospective observational study using data from donors who attended during the period analyzed. The automated chemiluminescence technique was used to detect anti-HTLV-1/2 antibodies.

**Results:**

A total of 60,568 donor samples were analyzed, of which 63 were positive, with a prevalence of 0.10% (95% confidence interval - 95%CI 0.08; 0.13) of HTLV-1/2 infection. The highest frequency (0.16%; 95%CI 0.10; 0.25) of infection was detected in 2020. Among donors with HTLV-1/2 infection, the following predominated: female, age group 31-45 years, brown skin color, high school education, and employment relationship in the private sector. The screening for HTLV-1/2 coinfection with other pathogens potentially associated with transfusion was positive for hepatitis B (n=5), syphilis (n=5), HIV (n=2), and hepatitis C (n=2). Based on the 95%CI analysis, no difference was observed between the ABO phenotypes of donors with positive HTLV-1/2 serology in the analyzed data.

**Conclusion:**

The prevalence of HTLV-1/2 observed in the study sample is similar to that reported by blood centers in southeastern Brazil, but higher than that reported by blood centers in the south. This result highlights the need for population-based epidemiological studies to identify the prevalence and distribution of HTLV-1/2 infection in Mato Grosso.

## Resumen

**Objetivos:**

Describir la prevalencia de la infección por el virus linfotrópico T humano tipos I y 2 (HTLV-1/2) entre los donantes de sangre del Centro de Sangre de Mato Grosso entre enero de 2018 y agosto de 2021, así como informar sobre las características demográficas y las coinfecciones identificadas en los donantes infectados.

**Métodos:**

: Se trata de un estudio observacional descriptivo y retrospectivo que utiliza datos de los donantes que acudieron durante el periodo analizado. Se utilizó la técnica automatizada de quimioluminiscencia para detectar anticuerpos anti-HTLV-1/2.

**Resultados:**

Se analizaron un total de 60 568 muestras de donantes, de las cuales 63 fueron positivas, con una prevalencia del 0,10 % (intervalo de confianza del 95 %: 0,08; 0,13) de infección por HTLV-1/2. La frecuencia más alta (0,16 %; IC del 95 %: 0,10; 0,25) de infección se detectó en 2020. Entre los donantes con infección por HTLV-1/2, predominaron los siguientes: mujeres, grupo de edad de 31 a 45 años, color de piel morena, educación secundaria y relación laboral en el sector privado. El cribado de la coinfección por HTLV-1/2 con otros patógenos potencialmente asociados a la transfusión dio positivo para hepatitis B (n = 5), sífilis (n = 5), VIH (n = 2) y hepatitis C (n = 2). Según el análisis del IC del 95 %, no se observaron diferencias entre los fenotipos ABO de los donantes con serología positiva para HTLV-1/2 en los datos analizados.

**Conclusión:**

La prevalencia de HTLV-1/2 observada en la muestra del estudio es similar a la reportada por los centros de transfusión sanguínea del sureste de Brasil, pero superior a la reportada por los centros de transfusión sanguínea del sur. Este resultado destaca la necesidad de realizar estudios epidemiológicos basados en la población para identificar la prevalencia y la distribución de la infección por HTLV-1/2 en Mato Grosso.

**Ethical aspects**

This research respected ethical principles, having obtained the following approval data:

Research ethics committee: Hospital Universitário Júlio Müller

Opinion number: 2891623

Approval date: 13/9/2018

Certificate of submission for ethical appraisal: 97198118.8.0000.5541

Research ethics committee: Secretaria de Estado de Saúde de Mato Grosso

Opinion number: 3393190

Approval date: 14/6/2019

Certificate of submission for ethical appraisal: 97198118.8.3001.5164

Informed consent record: Obtained from all participants prior to data collection.

## Introduction

Human T-lymphotropic virus types 1 and 2 (HTLV-1/2), a member of the Retroviridae family and the Deltaretrovirus genus, was the first retrovirus isolated in humans ^1^. In 2023, an estimated 5 to 10 million people worldwide were infected with HTLV-1 ^2^, with endemic regions of infection in Japan, Central Africa, Melanesia, the Caribbean, and South America ^3^. Brazil is the country with the highest number of HTLV-1 carriers in Latin America, with approximately 2 million people infected in 2021, primarily located in the North and Northeast regions of the country ^4^.

In 2023, the prevalence of HTLV-1 infection among blood donors ranged from 0.03% to 0.48% in Santa Catarina and Bahia, respectively ^2^. In Mato Grosso, a prevalence of 0.2% of HTLV-1/2 infection was identified in puerperal women in 2009 ^5^. Also in the Central-West region, the prevalence of HTLV was 0.017% among blood donors in Mato Grosso do Sul in 2023 ^6^ and 0.09% in Goiás in 2024 ^7^. Across the country, there has been an increase in the prevalence of HTLV-I/2 in groups of patients with hepatitis B and C viruses, human immunodeficiency virus (HIV), and tuberculosis ^8^ ^,^ ^9^ ^,^ ^10^ ^,^ ^11^ ^,^ ^12^ ^,^ ^13^ ^,^ ^14^. 

HTLV-1 is mainly detected in CD4+ T lymphocytes, and HTLV-1 in CD8+ T lymphocytes ^6^. Although HTLV-2 is not directly associated with pathologies, HTLV-1 is the etiological agent of adult T-cell leukemia and tropical myelopathy/spastic paraparesis, resulting in high morbidity and mortality, as well as conditions of immune hypersensitivity such as arthritis, fibromyalgia, and uveitis. There is no effective therapy available for the treatment of severe cases associated with HTLV-1 ^7^ ^,^ ^8^ ^,^ ^9^ ^,^ ^10^ ^,^ ^11^ ^,^ ^12^. HTLV-1/2 infection has been a notifiable disease in Brazil since 2024 and is mainly associated with unprotected sex and illicit intravenous drug use ^9^. 

Prevention of HTLV-1/2 transmission through blood transfusions is achieved through immunoenzymatic and agglutination techniques based on the presence of anti-HTLV-1/2 antibodies in blood samples from donors at blood banks in Brazil ^14^ ^,^ ^15^. Nevertheless, diagnostic confirmation of HTLV-1/2 requires more sensitive techniques that detect viremia, making it necessary to complement with molecular tests ^16^ ^,^ ^17^. 

The Mato Grosso Blood Center is a state public health unit that directly assists in planning and implementing state public health policies, in addition to being responsible for the supply and storage of blood on a statewide scale ^17^. This study aimed to describe the prevalence of HTLV-1/2 infection among blood donors at the Mato Grosso Blood Center between January 2018 and August 2021, and to report the demographic characteristics and coinfections identified in infected donors.

## Methods

Design 

This was a retrospective, descriptive, observational study using data from blood donors at the Mato Grosso Blood Center, collected from January 2018 to August 2021. 

Samples and study location

All blood samples from donors analyzed in this study were collected and processed simultaneously with the donation process at the Mato Grosso Blood Center located in Cuiabá. After laboratory screening, all samples were stored at -86ºC. Those with positive serology for HTLV-1/2 were retained in the blood center’s serology laboratory for confirmatory retesting.

Participants

The participants were all blood donors from the Mato Grosso Blood Center, based in Cuiabá, and from all collection and transfusion units located in the most populous municipalities of Mato Grosso, who made their donations between January 2018 and August 2021. 

Variables

For this analysis, information was obtained on the year of donation, age, sex, race/skin color, marital status, education, professional status, ABO phenotype/Rh factor, and infections detected in donors.

Data sources and measurement

The data were obtained from the Hemovida system of the Department of Information and Informatics of the Unified Health System, respecting confidentiality in accordance with specific regulations in the area. The Hemovida system is available from: http://siab.datasus.gov.br/DATASUS/index.php?area=060503.

Patient serum was tested for anti-HTLV antibodies by chemiluminescence using the Alinity HTLV-1/2 kit (Abbott, GmbH), as well as for anti-HBc, Syphilis Tp, HIV Ag/Ab combo, and anti-HCV, all according to the manufacturer’s instructions. Anti-HBc is an antibody that targets the viral core antigen of the hepatitis B virus-a qualitative, visual reading, *in vitro* immunoassay was used to detect antibodies against *Treponema pallidum*. The chemiluminescent microparticle immunoassay was used for the simultaneous qualitative detection of HIV p24 antigen and antibodies against human immunodeficiency virus type 1 and/or type 2 (HIV-1/HIV-2). A serological test was used to detect antibodies against the hepatitis C virus (anti-HCV) in the blood of donors. The screening tests were performed in singlicate, and samples with optical density values up to 20.0% below the detection threshold of 1 were considered reactive. Reactive samples were retested and confirmed using the same methodology.

Statistical methods 

Quantitative variables were categorized and, along with the qualitative ones, described by their absolute and relative frequency distributions. The prevalence of HTLV-1/2 infection was calculated by dividing the number of donors who tested reactive for anti-HTLV-1/2 by the total number of donors screened during the analyzed period. 

All relevant proportions were presented with their respective 95% confidence intervals (95%CI). Data were organized in Microsoft Excel spreadsheets and analyzed using Stata 12.0 statistical software (Stata Corp, California, United States). The Quantum Geographic Information System software, version 2.18.26, was used to describe the spatial distribution of donors infected with HTLV-1/2 in Mato Grosso. 

## Results

**Figure 1.**
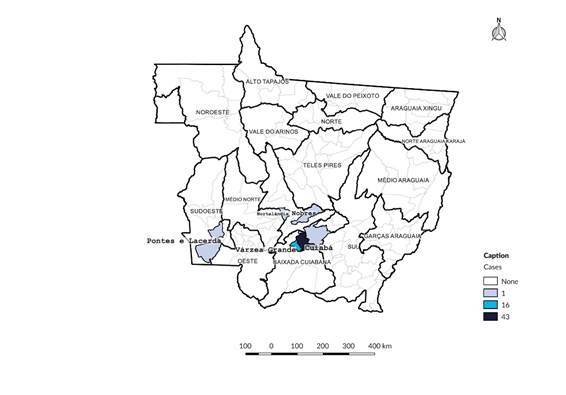
Spatial description of blood donors analyzed in the laboratory screening process with positive serology for human T-lymphotropic virus types 1 and 2 in relation to Cuiabá (n=43), Várzea Grande (n=16), Nobres (n=1), Nortelândia (n=1), Chapada dos Guimarães (n=1), and Pontes e Lacerda (n=1). Mato Grosso, 2018-2021 (n=63)

Data from 60,568 blood donors were analyzed, among whom 63 tested positive, resulting in a prevalence of 0.10% (95%CI 0.08; 0.13) of HTLV-1/2 infection. The highest frequency (0.16%; 95%CI 0.10; 0.25) of infection was detected in 2020. In contrast, the year 2019 showed the lowest prevalence of the analyzed period, with 0.05% of donors. A 44.4% increase in prevalence was observed in 2021 compared to 2018 (Table 1). These donors were predominantly recruited in Cuiabá, Várzea Grande, Pontes e Lacerda, Chapada dos Guimarães, Nortelândia, and Nobres, located in the central-southern and western regions of Mato Grosso (Figure 1).

**Table 1:** Distribution of samples and 95% confidence intervals (95%CI) for the overall blood donor population and those reactive for human T-lymphotropic virus (HTLV). Mato Grosso, 2018-2021 (n=60,568)

Year	Donors	Positive n (%)	Negative n (%)	% (IC95%)
2018	15917	15 (0.09)	15,902 (99.91)	0.09 (0.05; 0.16)
2019	17503	9 (0.05)	17,494 (99.95)	0.05 (0.02; 0.10)
2020	12949	21 (0.16)	12,928 (99.84)	0.16 (0.10; 0.25)
2021	14199	18 (0.13)	14,181 (99.87)	0.13 (0.07; 0.20)

During the screening process of HTLV-1/2-positive donors, 36 returned for retesting, and 26 of them confirmed the previous result (Table 2). Among the 63 donors who tested positive for HTLV-1/2, most were female, identified as having brown skin color, completed high school, and had employment relationships in the private sector (Table 3). 

**Table 2:** Confirmation of blood donor samples with reactive, gray zone, and unconfirmed results, close to the detection limit, for human T-lymphotropic virus. Mato Grosso, 2018-2021 (n=36)

Status	n
Confirmed reactive	
	
Reactive	26
Gray zone	5
Unconfirmed	
	
Near the detection threshold	5
Total	36

**Table 3:** Description of the demographic characteristics of blood donors infected with human T-lymphotropic virus (HTLV). Mato Grosso, 2018-2021 (n=63)

Characteristic		n
Sex	Male	30 (47.6)
	Female	33 (52.4)
Age group (years)	18-30	17 (27.1)
	31-45	34 (53.9)
	46-60	12 (19.0)
Marital Status	Married	22 (35.0)
	Single	30 (47.6)
	Other	11 (17.4)
Race/skin color	Brown	33 (52.4)
	Black	11 (17.4)
	White	19 (30.2)
Education level	None	-
	Complete elementary school	9 (14.3)
	Complete high school	37 (58.7)
	Superior education	17 (27.0)
Occupation	Self-employed professional or freelancer	4 (6.3)
	Retired or receiving social benefits	4 (6.3)
	Homemaker	5 (7.9)
	Private sector employee	18 (28.6)
	Public servant	15 (23.8)
	Unemployed	1 (1.7)
	Other	16 (25.4)

It was found that 34 donors had the O+ blood phenotype, 19 had the A+ phenotype, 2 had the A−phenotype, 4 had the B+ phenotype, 3 had the O−phenotype, and 1 had the B−phenotype. According to the 95%CI analysis, no difference was observed between ABO phenotypes among donors with positive HTLV-1/2 serology in the analyzed data (Table 4). 

Coinfection with other pathogens potentially associated with transfusion was observed in donors with HTLV-1/2 infection: hepatitis B virus (n=5), syphilis (n=5), HIV (n=2), and hepatitis C virus (n=2) (Table 5).

**Table 4:** Distribution^a^ of frequency and 95% confidence intervals (95%CI) of blood donors (n=60,568) infected with human T-lymphotropic virus(HTLV-1/2) according to ABO/Rh phenotypes. Mato Grosso, 2018-2021 (n=63)

ABO phenotype and Rh factor	Donors n	Reactive HTLV-1/2 n	% (95%CI)
A^+^	16788	19	0.113 (0.068; 0.177)
A^-^	2307	2	0.867 (0.010; 0.313)
B^+^	6518	4	0.061 (0.017; 0.157)
B^-^	722	1	0.138 (0.004; 0.769)
O^+^	28258	34	0.120 (0.083; 0.168)
O^-^	3961	3	0.076 (0.016; 0.221)
AB^+^	1813	-	-
AB^-^	201	-	-

**Table 5:** Coinfection markers with seropositivity for human T-lymphotropic virus (HTLV) among donors with reactive screening tests for anti-HBc, syphilis TP, HIV Ag/Ab combo, and anti-HCV. Mato Grosso, 2018-2021 (n=14)

Markers	n (%)
Anti-HBc^a^	5
Syphilis TP^b^	5
HIV Ag/Ab combo^c^	2
Anti-HCV ^d^	2

^a^Antibody against hepatitis B core antigen; ^b^Qualitative in vitro immunoassay with visual reading, used to detect *Treponema pallidum* antibodies in human serum; ^c^Automated qualitative in vitro detection of HIV p24 antigen and antibodies to human immunodeficiency virus type 1 and/or type 2 (HIV-1/HIV-2); ^d^Anti-HCV is a serological test that detects the presence of antibodies against hepatitis C virus (HCV) in the blood.

## Discussion

In this descriptive study, data from 60,568 blood donors at the Mato Grosso Blood Center were evaluated between January 2018 and August 2021. The estimated prevalence of HTLV-1/2 infection was 0.10%, similar to those reported for Mato Grosso do Sul ^6^ and Goiás ^7^, both located in the Central-West region, and which also evaluated blood donor populations. 

It is the first study to assess the prevalence and characteristics of donors deemed ineligible due to HTLV-1/2-positive results during the laboratory screening process at the Mato Grosso Hemocenter. Since 1993, the Brazilian Ministry of Health has mandated serological screening for HTLV-1/2 among blood donors. Anti-HTLV-1/2 screening in blood donors is a key strategy for ensuring transfusion safety, promoting quality public health, and contributing to the determination of epidemiological indicators of the infection ^4^.

Investigation of HTLV-1/2 infection in blood donors has been addressed in various studies, with proportions ranging from 0.08% in France to 0.20% in the United States and 0.29% in Canada ^19^ ^,^ ^20^ ^,^ ^21^ ^,^ ^22^ ^,^ ^23^. In Brazil, studies conducted with blood donors from different regions also show low prevalence rates, ranging from 0.02% in Mato Grosso do Sul ^6^, 0.03% in Santa Catarina ^21^, 0.04% in Paraná ^23^, 0.08% in Minas Gerais, 0.10% in São Paulo, and 0.13% in Amazonas ^7^. However, higher prevalence rates have been reported in the general population in the Northeast and North regions, with 0.48% in Bahia and 0.46% in Acre ^7^. The wide variation in HTLV-1/2 prevalence across different locations may be related to the demographic and behavioral characteristics of the studied populations, as well as differences in the policies implemented and screening tests used for these blood donors ^6^ ^,^ ^16^.

The Mato Grosso Blood Center is located in Cuiabá, the state capital, which is home to the largest population among the state’s 142 municipalities. Mato Grosso is characterized by its vast territorial area, low population density, and proximity to international borders. Municipalities in the Cuiabá lowlands were predominantly represented among HTLV-1/2-positive donors, which may be related to a higher transmission risk and social behavior patterns similar to those observed in large urban centers in Brazil. The greater likelihood of many people living in small areas, influenced by the sociocultural and demographic composition of the population, increases the risk of sexually transmitted infections ^24^. Only one case of HTLV-1/2 infection was identified in Pontes e Lacerda, located in the Vale do Guaporé region on the border with Bolivia, which may be related to the low Human Development Index and high poverty rate in that area ^25^.

The findings showed a predominance of HTLV-1/2 infection in 2020 and 2021, during the COVID-19 pandemic, compared to the pre-pandemic period. In contrast, a study conducted in South America reported a decrease in HTLV-1/2 infection in the period from 2020 to 2022 compared to 2018 to 2019 ^26^. 

Although variation in HTLV-1/2 infection was observed across the analyzed years, the results are consistent with other studies in the same population ^18^ ^,^ ^19^ ^,^ ^20^ ^,^ ^21^ ^,^ ^22^ ^,^ ^23^ ^,^ ^24^ ^,^ ^25^ ^,^ ^26^ ^,^ ^27^. These findings point to differences in the methodologies used, such as the use of molecular confirmatory tests or techniques other than those employed in serological screening. 

Chemiluminescence assays are reliable for detecting antibodies with high sensitivity and specificity in HTLV-1/2 serological screening ^24^. A small portion of donors did not have HTLV-1/2 infection confirmed upon retesting. In low-risk populations such as blood donors, serological screening may have a low predictive value ^25^ ^,^ ^26^ ^,^ ^27^ ^,^ ^28^ ^,^ ^29^. Inconclusive results may be related to several factors, including the seroconversion period, the use of HTLV strains different from those in testing kits, and low antibody titers at the time of analysis ^30^.

A single study reported a 0.2% seropositivity for HTLV-1/2 among postpartum women in Mato Grosso ^5^. These data support previous studies that indicated a higher prevalence of HTLV-1/2 infection among women, as well as a greater risk of sexual transmission, which increases with age ^2^ ^,^ ^3^ ^,^ ^31^.

Among the most clinically relevant HTLV-1/2 coinfections are those associated with HIV-1, hepatitis B and C viruses, and other sexually transmitted infections such as syphilis ^3^. HTLV-1/2 infection and other serological markers of viral infections were also investigated, providing important insights for the clinical management of blood donors. The results showed that among donors infected with HTLV-1/2, some also had antibodies indicating syphilis and hepatitis B and C infections, possibly because the study addressed only blood donors, who typically have limited exposure and a lower expected risk for sexually transmitted infections ^2^ ^,^ ^13^ ^,^ ^14^ ^,^ ^19^ ^,^ ^29^ ^,^ ^30^ ^,^ ^31^ ^,^ ^32^ ^,^ ^33^.

The lack of association between HTLV-1/2 infection and ABO/Rh phenotype among blood donors is consistent with other reports. However, previous studies have indicated higher prevalence rates among individuals with blood type AB and suggested a protective effect against infection for blood type A donors ^32^ ^,^ ^33^ ^,^ ^34^ ^,^ ^35^.

One limitation of this study was the need to update the Mato Grosso Hemocenter’s system, which hindered the generation of reports and data collection. Additionally, the absence of donors for the collection of a second sample limited the provision of more comprehensive data. 

In summary, the prevalence of HTLV-1/2 infection among blood donors at the Mato Grosso Hemocenter was low, and the distribution of demographic and clinical characteristics was similar to that reported in the literature ^34^ ^,^ ^35^. Collaborative studies and the future exchange of information between different regions of the country will help understand the epidemiology of HTLV-1/2 in Mato Grosso.
