# Supplementary material for: Prevalence of human T-cell lymphotropic virus in blood donor screening at the Blood Center: a descriptive study, Mato Grosso, 2018-2021
Source: Epidemiol Serv Saude. 2025 Feb 16;35:e20240799. doi: 10.1590/S2237-96222026v35e20240799.en (PMC12917950; doi:10.1590/S2237-96222026v35e20240799.en)
Supplement: Translation

# Translation: Prevalência do vírus linfotrópico de células T humanas em triagem de doadores de sangue no Hemocentro: estudo descritivo, Mato Grosso, 2018-2021

**DOI:** 10.1590/S2237-96222026v35e20240799.pt

**Authors:** Pennsylvania Marinho Borralho, Eduarda Pavan, Francisco Kennedy Scofoni Faleiros de Azevedo, Cor Jesus Fernandes Fontes, Anmony Borralho de Figueiredo, Wagner Izidoro de Brito, Fernanda Rizzieri Yamanaka, Ruberlei Godinho de Oliveira

**Affiliations:** 1 Hemocentro de Mato Grosso, Secretaria de Estado de Saúde de Mato Grosso, Cuiabá, MT, Brasil; 2 Universidade Federal de Mato Grosso, Laboratório de Virologia, Cuiabá, MT, Brasil; 3 Universidade Federal de Mato Grosso, Cuiabá, MT, Brasil; 4 Secretaria de Estado de Saúde de Mato Grosso, Cuiabá, MT, Brasil; 5 Hospital Universitário Júlio Müller, Cuiabá, MT, Brasil

**Keywords:** Deltaretrovirus, Prevalência, Demografia, Banco de Sangue, Estudos Soroepidemiológicos

## Abstract

**Objetivos:**

Descrever a prevalência de infecção pelo vírus linfotrópico de células T humanas tipos I e II (HTLV I/II) entre doadores de sangue do Hemocentro de Mato Grosso entre janeiro de 2018 e agosto de 2021, bem como relatar as características demográficas e as coinfecções identificadas nos doadores infectados.

**Métodos:**

Tratou-se de estudo observacional descritivo e retrospectivo com dados dos doadores que compareceram no período analisado. Utilizou-se a técnica de quimiluminescência automatizada para detecção de anticorpos anti-HTLV I/II.

**Resultados:**

Analisaram-se 60.568 amostras de doadores, das quais 63 resultaram positivas, com prevalência de 0,10% (intervalo de confiança de 95% - IC95% 0,08; 0,13) de infecção por HTLV I/II. Maior frequência (0,16%; IC95% 0,10; 0,25) de infecção foi detectada em 2020. Entre os doadores com infecção por HTLV I/II, predominaram: sexo feminino, faixa etária de 31-45 anos, raça/cor da pele parda, escolaridade de ensino médio completo e vinculação profissional com a iniciativa privada A pesquisa de coinfecção HTLV I/II com outros patógenos potencialmente associados à transfusão foi positiva para hepatite B (n=5), sífilis (n=5), HIV (n=2) e hepatite C (n=2). Pela análise do IC95%, não se observou diferença entre os fenótipos ABO dos doadores com sorologia positiva para HTLV I/II nos dados analisados

**Conclusão:**

A prevalência de HTLV I/II observada na amostra estudada é semelhante à reportada por hemocentros brasileiros do Sudeste, porém superior à reportada por hemocentros do Sul. Esse resultado ressalta a necessidade estudos epidemiológicos populacionais para a identificação da prevalência e distribuição da infecção pelo HTLV I/II em Mato Grosso.

**Aspectos éticos**

Esta pesquisa respeitou os princípios éticos, obtendo os seguintes dados de aprovação:

Comitê de ética em pesquisa: Hospital Universitário Júlio Müller

Número do parecer: 2.891.623

Data de aprovação: 13/9/2018

Certificado de apresentação de apreciação ética: 97198118.8.0000.5541

Comitê de ética em pesquisa: Secretaria de Estado de Saúde de Mato Grosso

Número do parecer: 3.393.190

Data de aprovação: 14/6/2019

Certificado de apresentação de apreciação ética: 97198118.8.3001.5164

Registro do consentimento livre e esclarecido: Obtido de todos os participantes antes da coleta.

Introdução 

O vírus linfotrópico de células T humanas tipos I e II (HTLV I/II), família *Retroviridae*, gênero *Deltaretrovirus*, foi o primeiro retrovírus isolado em humanos ^1^. Em 2023, estimou-se que 5 a 10 milhões de pessoas estivessem infectadas com HTLV I no mundo ^2^, com regiões endêmicas de infecção no Japão, na África Central, na Melanésia, no Caribe e na América do Sul ^3^. O Brasil é o país com maior número de portadores de HTLV I na América Latina, apresentando aproximadamente 2 milhões de pessoas infectadas em 2021, distribuídos principalmente nas regiões Norte e Nordeste do país ^4^.

Em 2023, as prevalências de infecção por HTLV entre doadores de sangue variaram entre 0,03% e 0,48% em Santa Catarina e na Bahia, respectivamente ^2^. Em Mato Grosso, foi identificada a prevalência de 0,2% de infecção por HTLV I/II em puérperas em 2009 ^5^. Também no Centro-Oeste, houve prevalências de HTLV de 0,017% entre os doadores de sangue de Mato Grosso do Sul em 2023 ^6^ e de 0,09% de Goiás em 2024 ^7^. No país, tem sido observado aumento da prevalência de HTLV-I/II em grupos de pacientes com os vírus das hepatites B e C e da imunodeficiência humana (HIV), e com tuberculose ^8^ ^,^ ^9^ ^,^ ^10^ ^,^ ^11^ ^,^ ^12^ ^,^ ^13^ ^,^ ^14^. 

O HTLV I é detectado principalmente em linfócitos T CD4^+^, e o HTLV II, em linfócitos T CD8^+ (^ ^6^. Apesar de o HTLV II não estar associado diretamente a patologias, o HTLV I é o agente etiológico da leucemia de células T do adulto e da mielopatia/paraparesia espástica tropical, resultando em alta morbidade e mortalidade, além de condições de hipersensibilidade imunológica como artrite, fibromialgia e uveíte. Não existe terapia eficaz disponível para o tratamento de casos graves associados ao HTLV I ^7^ ^,^ ^8^ ^,^ ^9^ ^,^ ^10^ ^,^ ^11^ ^,^ ^12^. A infecção por HTLV I/II é uma doença de notificação compulsória desde 2024 no Brasil, e está associada, principalmente, à prática de relações sexuais não protegidas e ao uso de drogas endovenosas ilícitas ^9^. 

A prevenção da transmissão transfusional de HTLV I/II é realizada por técnicas imunoenzimáticas e de aglutinação baseadas na presença de anticorpos anti-HTLV I/II em triagens de amostras de sangue dos doadores em hemocentros do Brasil ^14^ ^,^ ^15^. No entanto, a confirmação diagnóstica do HTLV I/II requer técnicas mais sensíveis e que detectem a viremia, tornando necessária a complementação com testes moleculares ^16^ ^,^ ^17^. 

O Hemocentro de Mato Grosso é uma unidade estadual de saúde pública que auxilia diretamente no planejamento e na implementação de políticas de saúde pública do estado, além de ser responsável pela oferta e pelo armazenamento do sangue em escala estadual ^17^. Este estudo teve como objetivos descrever a prevalência de infecção de HTLV I/II entre doadores de sangue do Hemocentro de Mato Grosso entre janeiro de 2018 e agosto de 2021, bem como relatar as características demográficas e as coinfecções identificadas nos doadores infectados.

Métodos 

Delineamento 

Tratou-se de estudo observacional descritivo retrospectivo com dados de doadores de sangue do Hemocentro de Mato Grosso, coletados de janeiro de 2018 a agosto de 2021. 

Amostras e local do estudo

Todas as amostras de sangue dos doadores analisados neste estudo foram coletadas e processadas simultaneamente ao processo de doação no Hemocentro de Mato Grosso situado em Cuiabá. Após a triagem laboratorial, todas as amostras foram armazenadas a -86ºC. Aquelas com sorologia positiva para HTLV I/II foram retidas no laboratório de sorologias do hemocentro, para retestagem confirmatória.

Participantes

Os participantes foram todos os doadores de sangue do Hemocentro de Mato Grosso, com sede em Cuiabá, e de todas as unidades de coleta e transfusão localizadas nos municípios mais populosos de Mato Grosso, que fizeram suas doações entre janeiro de 2018 e agosto de 2021. 

Variáveis

Para esta análise, foram obtidas informações sobre o ano da doação, a idade, o sexo, a raça/cor da pele, o estado civil, a escolaridade, o vínculo profissional, o fenótipo ABO/fator Rh e as infecções detectadas nos doadores.

Fontes de dados e mensuração

Os dados foram obtidos do sistema Hemovida do Departamento de Informação e Informática do Sistema Único de Saúde, respeitando a confidencialidade de acordo com regulamentações específicas da área. O sistema Hemovida está disponível em: http://siab.datasus.gov.br/DATASUS/index.php?area=060503.

O soro dos pacientes foi submetido à detecção de anticorpos anti-HTLV por quimiluminescência utilizando o kit Alinitys HTLV I/II (Abbott, GMBH), anti-HBc, Syphilis Tp, HIV Ag/Ab combo e anti-HCV, de acordo com as orientações do fabricante. O anti-HBc é o anticorpo contra o antígeno do core viral do vírus da hepatite B. O imunoensaio qualitativo, de leitura visual, *in vitro*, foi usado para detectar anticorpos contra *Treponema pallidum*. O imunoensaio de micropartículas por quimioluminescência foi utilizado para a detecção qualitativa simultânea do antígeno HIV p24 e de anticorpos contra o vírus da imunodeficiência humana tipo 1 e/ou tipo 2 (HIV 1/HIV 2). Foi utilizado exame sorológico para detecção de anticorpos contra o vírus da hepatite C (anti-HCV) no sangue dos doadores de sangue. Os testes de triagem foram realizados em simplicata, considerando-se reagente amostras com valores de densidade óptica até 20,0% menor que o valor de limite de detecção=1. Amostras reagentes seguiram para repetição e confirmação pela mesma metodologia.

Métodos estatísticos 

Variáveis quantitativas foram categorizadas e, juntamente com as qualitativas, foram descritas em suas distribuições de frequências absoluta e relativa. A prevalência de infecção pelo HTLV I/II foi calculada pela divisão do número de doadores com resultado reagente na pesquisa anti-HTLV I/II pelo total de doadores triados no período analisado. 

Todas as proporções relevantes foram apresentadas com respectivo intervalo de confiança 95% (IC95%). Os dados foram organizados em planilha Microsoft Excel e analisados pelo pacote estatístico Stata 12.0 (Stata Corp, Califórnia, Estados Unidos). Utilizou-se o software Quantum Geographic Information System na versão 2.18.26 para a descrição espacial dos doadores com infecção pelo HTLV I/II em Mato Grosso. 

Resultados 

Foram analisados os dados de 60.568 de doadores de sangue, dos quais 63 resultaram positivos, resultando em prevalência de 0,10% (IC95% 0,08; 0,13) de infecção por HTLV I/II. Maior frequência (0,16%; IC95% 0,10; 0,25) de infecção foi detectada em 2020. Em contraste, o ano de 2019 apresentou a menor prevalência do período analisado, com 0,05% dos doadores. Observou-se incremento de 44,4% na prevalência em 2021 quando esta foi comparada a 2018 (Tabela 1). Esses doadores foram predominantemente captados em Cuiabá, Várzea Grande, Pontes e Lacerda, Chapada dos Guimarães, Nortelândia e Nobres, localizados no centro-sul e oeste de Mato Grosso (Figura 1).

**Figura 1.** Descrição espacial dos doadores de sangue analisados no processo de triagem laboratorial com sorologia positiva para vírus linfotrópico de células T humanas tipos I e II com relação a Cuiabá (n=43), Várzea Grande (n=16), Nobres (n=1), Nortelândia (n=1), Chapada dos Guimarães (n=1) e Pontes e Lacerda (n=1). Mato Grosso, 2018-2021 (n=63)

**Tabela 1:** Distribuição de amostras e intervalos de confiança de 95% (IC95%) de doadores de sangue gerais e dos reagentes para o vírus linfotrópico de células T humanas. Mato Grosso, 2018-2021 (n=60.568)

Ano	Doadores	Positivo n (%)	Negativo n (%)	% (IC95%)
2018	15.917	15 (0,09)	15.902 (99,91)	0,09 (0,05; 0,16)
2019	17.503	9 (0,05)	17.494 (99,95)	0,05 (0,02; 0.10)
2020	12.949	21 (0,16)	12.928 (99,84)	0,16 (0,10; 0,25)
2021	14.199	18 (0,13)	14.181 (99,87)	0,13 (0,07; 0,20)

Durante o processo de triagem dos doadores com HTLV I/II positivo, 36 deles retornaram para retestagem, dos quais 26 confirmaram o resultado anterior (Tabela 2). Entre os 63 doadores com sorologia reativa para HTLV I/II durante o processo de triagem, predominaram doadores do sexo feminino, da raça/cor da pele parda, de ensino médio de escolaridade e com vínculo profissional na iniciativa privada (Tabela 3). 

**Tabela 2:** Confirmação de amostras de doadores de sangue com resultados reagentes, zona cinza e não confirmadas, próximo ao limite de detecção, para o vírus linfotrópico de células T humanas. Mato Grosso, 2018-2021 (n=36)

Status	n
Confirmada reagente	
	
	
Reagente	26
Zona cinza	5
Não confirmadas	
	
	
Próximo ao limite de detecção	5
Total	36

**Tabela 3:** Descrição das características demográficas dos doadores de sangue com infecção pelo vírus linfotrópico de células T humanas. Mato Grosso, 2018-2021 (n=63)

Característica		n (%)
Sexo	Masculino	30 (47,6)
	Feminino	33 (52,4)
Faixa etária (anos)	18-30	17 (27,1)
	31-45	34 (53,9)
	46-60	12 (19,0)
Estado civil	Casado	22 (35,0)
	Solteiro	30 (47,6)
	Outros	11 (17,4)
Raça/cor da pele	Parda	33 (52,4)
	Preta	11 (17,4)
	Branca	19 (30,2)
Escolaridade	Nenhuma	-
	Ensino fundamental	9 (14,3)
	Ensino médio	37 (58,7)
	Superior	17 (27,0)
Ocupação	Profissional liberal ou autônomo	4 (6,3)
	Aposentado ou em benefício	4 (6,3)
	Do lar	5 (7,9)
	Empregado de empresa privada	18 (28,6)
	Funcionário público	15 (23,8)
	Desempregado	1 (1,7)
	Outros	16 (25,4)

Constatou-se que 34 doadores pertenciam ao fenótipo sanguíneo O^+^; 19, ao grupo A^+^; 2, ao grupo A^-^; 4, ao grupo B^+^; 3, ao grupo O^-^; e 1, ao grupo B^-^. Pela análise do IC95%, não se observou diferença entre os fenótipos ABO dos doadores com sorologia positiva para HTLV I/II nos dados analisados (Tabela 4). 

A pesquisa de coinfecção HTLV I/II com outros patógenos potencialmente associados à transfusão foi positiva para o vírus da hepatite B (n=5), a sífilis (n=5), o HIV (n=2) e o vírus da hepatite C (n=2) (Tabela 5).

**Tabela 4:** Distribuição^a^ da frequência e intervalos de confiança de 95% (IC95%) de doadores de sangue (n=60.568) com infecção pelo vírus linfotrópico de células T humanas (HTLV I/II), segundo o fenótipo ABO/Rh. Mato Grosso, 2018-2021 (n=63)

Fenótipo ABO e fator Rh	Doadores n	HTLV I/II reagente n	% (IC95%)
A^+^	16.788	19	0,113 (0,068; 0,177)
A^-^	2.307	2	0,867 (0,010; 0,313)
B^+^	6.518	4	0,061 (0,017; 0,157)
B^-^	722	1	0,138 (0,004; 0,769)
O^+^	28.258	34	0,120 (0,083; 0,168)
O^-^	3.961	3	0,076 (0,016; 0,221)
AB^+^	1.813	-	-
AB^-^	201	-	-

**Tabela 5:** Marcadores de coinfecção que apresentaram soropositividade com a infecção pelo vírus linfotrópico de células T humanas nos testes de triagens positivas para anti-Hbc, Syphilis TP, HIV Ag/Ab combo e anti-HCV. Mato Grosso, 2018-2021 (n=14)

Marcadores	n
Anti-HBc^a^	5
Syphilis TP^b^	5
HIV Ag/Ab combo^c^	2
Anti-HCV ^d^	2

^a^Anticorpo contra o antígeno do core do vírus da hepatite B; ^b^Imunoensaio qualitativo in vitro, de leitura visual, usado para detectar anticorpos do *Treponema pallidum* no soro humano; ^c^Detecção qualitativa automática in vitro do antígeno p24 do HIV e anticorpos para o vírus da imunodeficiência humana tipo 1 e/ou tipo 2 (HIV-1/HIV-2); ^d^Anti-HCV é um exame sorológico que detecta a presença de anticorpos contra o vírus da hepatite C (HCV) no sangue.

Discussão 

Neste estudo descritivo, foram avaliados os dados de 60.568 doadores de sangue do Hemocentro de Mato Grosso entre janeiro de 2018 e agosto de 2021. A prevalência estimada de infecção pelo HTLV I/II foi 0,10%, semelhante às reportadas para Mato Grosso Sul ^6^ e Goiás ^7^, ambos pertencentes à região Centro-Oeste e que também avaliaram população de doadores de sangue. 

Este é o primeiro estudo que avaliou a prevalência e as características dos doadores inaptos por HTLV I/II positivo durante o processo de triagem laboratorial no Hemocentro de Mato Grosso. O Ministério da Saúde preconiza a realização obrigatória de triagem sorológica para HTLV I/II entre doadores de sangue desde 1993. A pesquisa do anti-HTLV I/II na triagem do doador de sangue é estratégia fundamental para garantir a segurança transfusional, promover saúde pública de qualidade e auxiliar na determinação de índices epidemiológicos da infecção ^4^.

A investigação da infecção de HTLV I/II em doadores de sangue tem sido abordada em diversos estudos, com proporções variando de 0,08% na França, 0,20% nos Estados Unidos e 0,29% no Canadá ^19^ ^,^ ^20^ ^,^ ^21^ ^,^ ^22^ ^,^ ^23^. No Brasil, estudos realizados com doadores de sangue de diferentes regiões mostram prevalências também baixas, que variaram de 0,02% em Mato Grosso do Sul ^6^, 0,03% em Santa Catarina ^21^, 0,04% no Paraná ^23^, 0,08% em Minas Gerais, 0,10% em São Paulo e 0,13% no Amazonas ^7^. Contudo, prevalências mais altas foram descritas na população geral no Nordeste e no Norte, com 0,48% na Bahia e 0,46% no Acre ^7^. A grande variação da prevalência de HTLV I/II entre diferentes localidades pode estar relacionada às características demográficas e comportamentais das populações estudadas, bem como às variações nas políticas implementadas e nos testes de triagem entre esses doadores de sangue ^6^ ^,^ ^16^.

O Hemocentro de Mato Grosso está localizado em Cuiabá, a capital. Esta concentra a maior população desse estado de 142 municípios. Mato Grosso é caracterizado por sua grande extensão, baixa densidade populacional e regiões de fronteira internacional. Os municípios da baixada cuiabana foram prevalentes entre os doadores positivos de HTLV I/II, que podem estar relacionado com maior risco de transmissão, com padrão de comportamento social semelhantes aos demais grandes centros das cidades brasileiras. A maior probabilidade de muitas pessoas vivendo em pequenas áreas influenciadas pela composição sociocultural e demográfica da população confere comportamento de risco para as infecções sexualmente transmissíveis ^24^. Apenas uma infecção pelo HLTV I/II foi identificada em Pontes e Lacerda, região do Vale do Guaporé, na divisa com a Bolívia, que pode estar relacionada ao baixo índice de desenvolvimento humano e ao alto índice de pobreza dessa região ^25^.

Os achados demonstraram que houve predomínio de infecção para HTLV-I/II em 2020 e 2021, período da pandemia de covid-19, em relação ao período pré-pandêmico. Em contrapartida, uma pesquisa realizada na América do Sul demonstrou a redução da infecção de HTLV I/II no período 2020-2022, quando comparado ao período 2018-2019 ^26^. 

Embora ocorra variação entre a infecção de HTLV I/II entre os anos analisados, os resultados são semelhantes aos de outros estudos na mesma população ^18^ ^,^ ^19^ ^,^ ^20^ ^,^ ^21^ ^,^ ^22^ ^,^ ^23^ ^,^ ^24^ ^,^ ^25^ ^,^ ^26^ ^,^ ^27^. Esses resultados indicam diferenças de metodologias utilizadas com realização de testes confirmatórios moleculares ou técnicas diferentes dos testes realizados na triagem sorológica. 

Ensaios de quimiluminescência são confiáveis para a detecção de anticorpos com alta sensibilidade e especificidade, na triagem sorológica do HTLV I/II ^24^. Uma pequena parcela de doadores não teve a infecção de HTLV I/II confirmada na segunda testagem. Em populações de baixo risco como doadores de sangue, triagens sorológicas podem ter valor preditivo baixo ^25^ ^,^ ^26^ ^,^ ^27^ ^,^ ^28^ ^,^ ^29^. Resultados inconclusivos podem ser relacionados a diferentes fatores, como período de soroconversão, cepas de HTLV diferentes das utilizadas pelos kits e presença de baixa titulação de anticorpos no momento da análise ^30^.

Um único estudo descreve 0,2% de soropositividade para o HTLV-I/II entre as mulheres puérperas em Mato Grosso ^5^. Estes dados corroboram estudos anteriores que indicaram maior prevalência de infecção por HTLV I/II entre as mulheres, bem como maior risco de transmissão pela via sexual, acentuado e relacionado ao aumento da idade ^2^ ^,^ ^3^ ^,^ ^31^.

Entre as coinfecções virais de maior relevância clínica para o HTLV I/II, destacam-se aquelas relacionadas ao HIV 1, hepatites causadas pelos vírus das hepatites B e C, além de outras infecções sexualmente transmissíveis como a sífilis ^3^. Infecção do HTLV I/II e outros marcadores sorológicos de infecções virais também foi investigada, fornecendo percepções importantes para o diagnóstico de manejo clínico dos doadores de sangue. Os resultados demonstraram que, entre os doadores infectados por HTLV I/II, alguns apresentaram anticorpos para a detecção da sífilis e dos vírus das hepatites B e C, possivelmente pelo fato de este estudo abordar apenas doadores de sangue, que são pessoas com poucas exposições e com esperado menor risco para infecção sexualmente transmissível ^2^ ^,^ ^13^ ^,^ ^14^ ^,^ ^19^ ^,^ ^29^ ^,^ ^30^ ^,^ ^31^ ^,^ ^32^ ^,^ ^33^.

A falta de associação entre infecção por HTLV I/II e fenótipo ABO/Rh entre os doadores de sangue estão de acordo com outros relatos, embora estudos anteriores tenham relatado taxas de prevalência mais altas entre indivíduos com tipo sanguíneo AB, com proteção à infecção aos doadores do tipo A ^32^ ^,^ ^33^ ^,^ ^34^ ^,^ ^35^.

Uma limitação deste estudo foi a necessidade de atualização do sistema do Hemocentro de Mato Grosso, o que dificultou a emissão de relatórios e a coleta de dados. Além disso, as ausências de doadores para a coleta da segunda amostra limitaram o fornecimento de dados mais abrangentes. 

Em síntese, a prevalência de infecção pelo HTLV I/II entre os doadores de sangue do Hemocentro de Mato Grosso foi baixa e com distribuição das características demográficas e clínicas semelhantes às já relatadas na literatura ^34^ ^,^ ^35^. A importância de estudos colaborativos e a troca de informações futuras entre diferentes regiões do país serão úteis para o entendimento da epidemiologia do HTLV I/II em Mato Grosso
